# Circular Business Model for Digital Health Solutions: Protocol for a Scoping Review

**DOI:** 10.2196/47874

**Published:** 2023-11-24

**Authors:** Camille Rønn, Andreas Wieland, Christiane Lehrer, Attila Márton, Jason LaRoche, Adrien Specker, Pascal Leroy, Daniel Fürstenau

**Affiliations:** 1 Department of Business IT IT University of Copenhagen Copenhagen Denmark; 2 Department of Operations Management Copenhagen Business School Copenhagen Denmark; 3 Department of Digitalization Copenhagen Business School Copenhagen Denmark; 4 Janssen Biologics The Janssen Pharmaceutical Companies of Johnson & Johnson Leiden Netherlands; 5 World Resources Forum St Gallen Switzerland; 6 Waste Electrical and Electronic Equipment Forum Brussels Belgium

**Keywords:** business model, circular economy, digital health solution, digital health, digital tool, digital, healthcare, life cycle, MedTech device, monitoring device, technology

## Abstract

**Background:**

The circular economy reshapes the linear “take, make, and dispose” approach and evolves around minimizing waste and recapturing resources in a closed-loop system. The health sector accounts for 4.6% of global greenhouse gas emissions and has, over the decades, been built to rely on single-use devices and deal with high volumes of medical waste. With the increase in the adoption of digital health solutions in the health care industry, leading the industry into a new paradigm of how we provide health care, a focus must be put on the amount of waste that will follow. Digital health solutions will shape health care through the use of technology and lead to improved patient care, but they will also make medical waste more complex to deal with due to the e-waste component. Therefore, a transformation of the health care industry to a circular economy is a crucial cornerstone in decreasing the impact on the environment.

**Objective:**

This study aims to address the lack of direction in the current literature on circular business models. It will consider micro, meso, and macro factors that would impact the operational validity of circular models using the digital health solutions ePaper label (medical packaging), smart wearable sensor (health monitoring devices), smart pill box (medication management), and endo-cutter (surgical equipment) as examples.

**Methods:**

The study will systematically perform a scoping review through a database and snowball search. We will analyze and classify the studies from a predetermined set of categories and then summarize them into an evidence map. Based on the review, the study will develop a 2D framework for businesses to follow or for future research to take a standpoint from.

**Results:**

Preliminarily, the review has analyzed 26 studies in total. The results are close to equally distributed among the micro (8/26, 31%), meso (10/26, 38%), and macro (8/26, 31%) levels. Circular economy studies emphasize several circular practices such as recycling (17/26, 65%), reusing (18/26, 69%), reducing (15/26, 58%), and remanufacturing (8/26, 31%). The value proposition in the examined business model is mostly dominated by stand-alone products (18/26, 69%) compared to product as a service (7/26, 27%), involving stakeholders such as health care professionals or hospitals (20/26, 77%), manufacturers (11/26, 42%), and consumers (9/26, 35%). All studies encompass societal (12/26, 46%), economic (23/26, 88%), and environmental (24/26, 92%) viewpoints.

**Conclusions:**

The study argues that each digital health solution would have to be accessed individually to find the optimal business model to follow. This is due to their differing life cycles and complexity. The manufacturer will need a layered value proposition, implementing several business models dependent on their respective product portfolios. The need to incorporate several business models implies an ecosystem perspective that is relevant to consider.

**International Registered Report Identifier (IRRID):**

DERR1-10.2196/47874

## Introduction

### Overview

A circular economy is a system that has been evolving in theory and practice for decades, with a core focus on minimizing waste and reprocessing products in a closed-loop system [1-4]. Grounded in a circular economy, a circular business model follows the same core fundament by emphasizing the creation and capture of value [2,5]. Businesses in resource-intensive industries, such as health care, can find a lot of value in implementing circular business models for their products to decrease their environmental footprints and become more resource efficient. Although circularity is not a mainstream model, several sectors have successfully started incorporating circular practices, especially in the utilities and consumer goods industries [6,7]. Yet with the advancement of circular models, health care seems to lag in the implementation of circular practices compared to other industries [8].

As global technological advancement continues, the health care industry has witnessed a surge in the adoption of digital health solutions across hospitals, health care professionals, and patients [9]. Given the increased production and use of digital health solutions, they have become a good use case for circular business models and could serve as a pivotal tool for reshaping the health care supply chain and business models. These intricate products are resource-intensive, comprising diverse materials such as metals, plastics, glass, and electronic components, all handled in the current linear waste management system. A waste management system refers to a strategy for how companies dispose of, reduce, and reuse waste [10,11]. Waste management in health care is often complex, especially in hospitals, with multiple streams of high volumes of waste generated daily [10-12]. Although several studies have delved into incorporating circular practices in hospital or home care waste management systems [10,11], research on a broader system-level viewpoint incorporating the supply chain, its stakeholders, and their interconnectedness is lacking [13]. Therefore, the main focus of this review will not be the patient but rather the system and its stakeholders. Nevertheless, as patient focus is at the core of all health care operations, this should be done while acknowledging and considering the role and importance of the patient in it.

The current literature landscape’s lack of consensus on how to develop a circular business model in health care does not arise due to disagreements on how circular waste management should be introduced but rather stems from a lack of knowledge and research on the topic. This conundrum could hint toward current circular business model solutions being too generalized and not considering the specificities of the health care industry and digital health to be implemented. Circularity must thus be implemented on a product- or industry-specific level to succeed [7].

**Figure 1:**
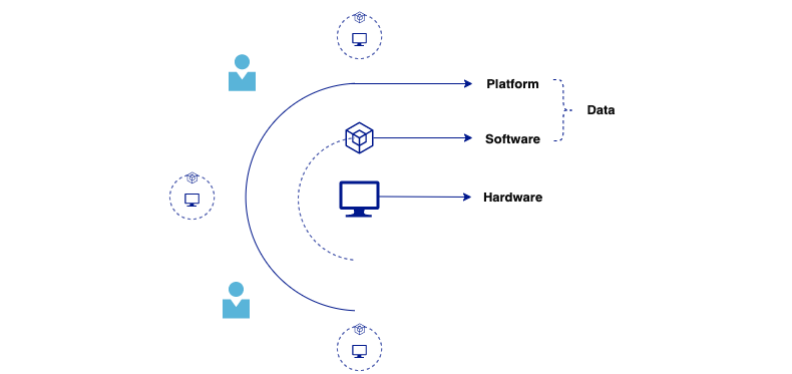
Illustration of components in a digital health solution.

For instance, 2 crucial characteristics of digital health solutions would make the implementation of a generic circular model fail. First, the term digital health “solution” is used on purpose instead of “device” because the product not only contains hardware but also software and data components (Figure 1). Apart from the circular practices of the solution’s hardware, we must also consider the waste of data, both in terms of the climate impact data storage has through embedded CO_2_ emissions due to electricity use and the continuous use as well as the repurposing of data. Even within digital health solutions, a distinction must also be made between the different types of life cycles the solutions have due to their varying use. Some digital health solutions never leave the hospital, in which case it can be easier to control their reprocessing, while other solutions are owned by the patients, which can leave uncertainties on take-back schemes in a circular business model. Second, the contamination risk of medical solutions adds an additional layer of regulatory and operational complexity that also must be considered in the operations of the circular model. You need to ensure the product does not spread or contain harmful elements impacting human health and the environment. The motivation behind developing an industry-specific model forms the purpose of this scoping review: to theoretically develop a framework that businesses can take as a standpoint [7]. This fills the call for research combining circularity and digital health in health care and bridging the research gaps by merging the concepts.

To develop concepts for tailored circular business models for digital health solutions, this scoping review examines existing circular models in the health care industry and of other similar products in other industries. To make the analysis concrete, 4 product categories will be used as examples: medical packaging (ePaper label), health monitoring devices (smart wearable sensor), medication management (smart pill box), and surgical equipment (endo-cutter). These 4 products are representative of medical versus nonmedical, as well as single-use versus recurrent-use products.

### Unit of Analysis

The unit of analysis in the scoping review is the circular business model. This is due to it being the core concept that motivates and justifies the performance of the study. The units of analysis can be broken down into circular and business models. Circularity will be defined through the concept of the 4 R’s (recycle, reuse, reduce, and remanufacture) and the MacArthur butterfly model [2] and hence refers to the concept of creating a closed-loop system where resources are remanufactured, reused, and recycled instead of being disposed of after use. The 4 R’s and the butterfly model both emphasize the importance of reducing waste and creating a circular economy. The second part of the unit of analysis, the business model, is a framework that describes how an organization creates, delivers, and captures value [14]. It is a conceptual tool that helps businesses articulate and communicate their strategy, operations, and revenue models in a concise and structured manner [5,14].

### Theoretical Boundaries

A set of theoretical boundaries will guide the scoping review. First, as the research on circular business models in health care is limited, the search does not solely focus on the health care industry but also related industries such as wellness. To avoid a scope that is too broad, it is important to narrow down the types of solutions to be examined and only include products that have similar components (Figure 1) or business models to a digital health solution. The study will therefore include specific types of products that contain hardware, software, or data and preferably are part of a highly regulated industry. Second, the study will not only take into account traditional business model factors such as revenue stream and value proposition but also the environmental and societal impact of the circular model being examined. This would involve a comprehensive analysis of the economic, environmental, and social benefits and costs of the circular model being proposed or implemented, as well as any potential trade-offs that may arise.

### Relevance

The scoping review will provide valuable information for future research in the circular economy as well as within the European Union (EU)–funded Digital Health in Circular Economy (DiCE) project and the stakeholders involved [15]. The adoption of circular economy principles by businesses developing digital health solutions has the potential to align with the United Nations’ Sustainable Development Goals (SDGs) [16]. The scoping review addresses SDG 3 on good health and well-being and SDG 9 on industry, innovation, and infrastructure [16]. Furthermore, the circular economy can also support economic growth and job creation. Lastly, the research is also relevant to SDG 12, as part of the business model comes from the value of returned materials, both in economic terms and in more environmental settings, to avoid depleting scarce resources [15].

## Methods

### Overview

The scoping review will follow a systematic framework and best practices developed by Arksey and O’Malley [17], Durach et al [18], and the Joanna Briggs Institute scoping review manual [19]. To establish guidelines for the data collection, the protocol is outlined below before conducting the review. The process and data collection will be structured around the following steps: identify the research question, determine the required characteristics of relevant studies, search strategy, study selection, synthesize the literature, and report the results.

### Identify the Research Question

The scoping review and its research question will be built around the unit of analysis and on the defined research boundaries listed. As the research question has an exploratory objective as well as an evaluation perspective on current circular business models, the following research question has been formulated: “How can circular business models be adapted and remodeled to digital health solutions?”

The research question will synthesize the literature on circular business models with a health care–oriented lens. To support the research question, a set of subquestions has been created to guide the analysis.

1. What are the barriers and enablers of implementing circular economy practices in the digital health market, and how can they be addressed? (This question will enlighten why traditional circular practices do not apply to the digital health market and where the existing models should be adapted.)
2. How are value and cost both negatively and positively redistributed in implementing a circular business model and impacting its key targets and performance indicators? (This question will discover how the business model of the solutions will change in terms of value. Value can be seen as financial, epistemic, ethical, service, and reputation.)
3. How would operating systems and data gathered by the solutions be affected positively or negatively by circularity? (This question focuses specifically on the software and data management parts of the solutions to ensure circularity is not only observed from the hardware perspective.)

### Determine the Required Characteristics of Relevant Studies

To identify a relevant set of studies, the following inclusion and exclusion criteria (Textbox 1) have been chosen.

**Inclusion and exclusion criteria.**

**Inclusion criteria**

- Studies focusing on one or more of the components in the solutions being hardware, software, and data
- Studies including health care or related industries such as fitness and wellness
- Studies including one or several stakeholders in a circular business model
- Studies illustrating environmental or societal impact by implementing circular business models

**Exclusion criteria**

- Studies that do not focus on economical, societal, or consumer outcome
- Studies published before the year 2010 [4,20]
- Non–peer-reviewed articles
- Editorials and commentaries
- Non-English studies

### Search Strategy

In the literature search, 2 search strategies will be implemented: keyword search and snowball search. The keyword search will be performed in Scopus and Web of Science. Furthermore, a separate cross-checking search in PubMed has been done to ensure the databases include studies within the industry of interest, health care. Scopus and Web of Science have been chosen for several reasons. First, they both cover interdisciplinary results, thus fitting the scope of the scoping review into circularity, business model, and health care. Second, the databases have similar search functions, making the standard search protocol easier to follow without too much adaptation from one database to another. Third, they are some of the biggest databases and have a broad reach. Following the research protocol, a set of search criteria and search strategies have been applied when searching in the mentioned databases.

The snowball search will be performed by screening the reference list of selected studies and their references. The dual search approach will help ensure a more comprehensive scoping review and provide a complete understanding of the topic. The keywords have been chosen by testing the number of hits for each keyword and using the snowball search technique to choose relevant keywords.

A total of 3 search queries have been created to enlighten the different layers of the research question (Table 1). One topic is the life cycle and supply chain of digital health solution. No relevant results appeared when searching for explicit terms of digital health. Therefore, the first search has a broader level and focuses on products in health care, wellness, fitness, or medicine and will be manually sorted based on whether they are or resemble digital health solutions. The second and third topics are the solution’s individual components, which are hardware, software, and data.

**Table 1:** Search queries.

Topic	Search query
Circular economy and processes	AB= (circul* OR “closed loop” OR “waste management” OR “Waste disposal”) AND AB= (“Business model*” OR “Operation*” OR “Value creation*” OR strateg*) AND AB= (“Supply chain*” OR “supply network” OR “process” OR “value chain” OR “life cycle” OR Logistic*) AND AB=(Healthcare OR Wellness OR fitness OR Medical))
Circular economy and hardware	((((AB=(circul* OR “losed loop” OR “waste management” OR “Waste disposal”)) AND AB=(“Business model*” OR “Operation*” OR “Value creation*” OR strateg*)) AND AB=(“Supply chain*” OR “supply network” OR “process” OR “value chain” OR “life cycle” OR Logistic* )) AND AB=(metal* OR plastic* OR e-waste OR “electronic waste” OR ceramics OR polymers OR glass OR composite))
Circular economy, data, and software	AB= (circul* OR “closed loop” OR “waste management” OR “Waste disposal” OR green OR sustain*) AND AB= (“Business model*” OR “Operation*” OR “Value creation*” OR strateg*) AND AB=(“Supply chain*” OR “supply network” OR “process” OR “value chain” OR “life cycle” OR Logistic* )) AND AB= (“information” OR data) AND (tech* OR platform OR Software OR system OR program*)

### Study Selection

In the study selection, all duplication of records is removed before a screening of the topic is performed. Furthermore, the inclusion and exclusion criteria will be used to reduce the final selection of studies. The results from the keyword search will be manually screened by 2 independent researchers to be able to compare and discuss the final study selection. The tool Rayyan (Qatar Computing Research Institute) will be used for this procedure. This method ensures the screening is less biased by the researchers’ different fields of expertise. In the data extraction process, the data will be inserted into the reference manager Zotero (Corporation for Digital Scholarship).

Both empirical qualitative and quantitative studies, as well as literature reviews, conceptual pieces, and modeling research, will be considered to enlighten the topic from different methodological perspectives. Table 2 has been created to guide the study selection.

**Table 2:** Patient or population, intervention, comparison, and outcomes (PICO) model.

Criteria	Description
Unit of analysis	Users of digital medical solutions or users of similar products
Intervention	Noninterventional study. The study will observe business models of a digital health solution.
Comparison	Circular versus linear business models^a^
Outcome	Positive and negative outcome are accepted

^a^Linear model refers to a “take-make-dispose” operational practice [21].

### Synthesize the Literature and Report the Results

The selected studies will be coded through an emerging and a priori coding structure to find relevant relationships between variables. With a priori code, we mean predetermined categories will be screened for when reporting the results. It will in addition extract relevant details on the studies such as title, author, publication details, and methods.

The results will be analyzed and summarized through a statistical view in tables and graphs as well as a narrative method. An evidence map will be created, with the set of categories that have been chosen below to categorize the data and find similarities and differences among the results to discuss (Table 3). The categories have been chosen to cover circular elements, characteristics of the unit of analysis, description of their business model, and their added value. At last, the results will be presented through both a descriptive analysis and a thematic analysis.

In addition to the categories chosen in the evidence map, the 3 levels, micro, meso, and macro, will be the structure of the discussion and help find the current research gaps in the literature.

**Table 3:** Evidence map categories described.

Categories and types			Description
**Circularity industry focus**			
	General perspective	Circularity of any relevant product or process	
	Health care perspective	Circularity within the health care industry and its products	
**Level of circular adoption**			
	Micro	Product and single firm level	
	Meso	Network of firms	
	Macro	Regional, national, or European Union level	
**Butterfly model**			
	Technical cycles	Nonbiodegradable materials such as metals. The main strategy is maintaining or reusing the materials	
	Biological cycles	Biodegradable materials such as biodegradable plastic	
**4 R’s**			
	Recycle	Collecting and processing materials into a new product instead of throwing them away	
	Reuse	Finding new ways to use products and materials to extend their life span and reduce waste	
	Reduce	Minimizing the use of resources, product, and packaging	
	Remanufacture	Rebuild or restore a product	
**Governance of the device**			
	Owned by consumer	The consumer has full ownership of the product and the use or disposal of it	
	Owned by intermediaries such as HCPs^a^ or hospitals	The organizations buy the product from the manufacturer and use it or lease or lend out the product for a certain period of time to the consumer	
	Owned by manufacturer	The manufacturer lends or leases out products to HCPs or patients	
	Not applicable	Governance of product not specified	
**Value creation for stakeholders**			
	Manufacturer	A company that produces and sells the product	
	Consumer	The end-client or patient actively using the products	
	Government	Regulating and overseeing health care policies and practices	
	Intermediaries such as HCPs or hospitals	Organizations that operate between the manufacturers and end consumers	
**Value impacts**			
	Societal	Actions or events that are harmful or beneficial on the society	
	Economical	Actions or events that lead to a decline or growth in economic prosperity	
	Environmental	Actions or events harmful or beneficial to natural environment	
**Digital health solutions**			
	Single-use devices	Devices that are used a single time and thereafter obsolete, such as syringes, needles, etc	
	Imaging	Devices with the purpose of creating scanning or images of patients’ body parts such as ultrasounds	
	Medical equipment	Devices used to monitor patients such as a wearable	
	Software	A computer system often used in collaboration with hardware, and can be used for diagnosing, monitoring, storing data, etc	
	In vitro diagnostics	Products used that come in contact with human tissue such as a glucose meter	
	Personal protective equipment	Worn by medical staff or used by patients as masks, gowns, and gloves	
	Surgical and laboratory instruments	Devices used in surgeries such as endocutters and endoscopes	

^a^HCP: health care professional.

### Ethical Considerations

This scoping review does not require ethical approval as it solely uses published articles with no research involving humans, medical records, patient information, or observations of public behaviors. Therefore, the term “not applicable” applies. Any deviations from this protocol will be disclosed in the following scoping review publication. Additionally, the outcomes will be disseminated in the research community, such as through conference participation.

## Results

### Overview

A preliminary description of the results has been performed. The results have been ranked according to the hierarchy of evidence to assess how likely they are to incorporate a systematic bias. There are 7/26 (27%) systematic reviews, 18/26 (69%) controlled studies, and 1/26 (4%) expert opinion. The results are well distributed among the 3 levels that will be analyzed: micro (8/26, 31%), meso (10/26, 38%), and macro (8/26, 31%). The reporting of results is divided into the unit of analysis, the circular business models, and the type of product analyzed in the studies.

### Unit of Analysis

#### Circular Economy

Most studies incorporate 2 or more R’s in their analysis (18/26, 69%). Reuse (18/26, 69%), recycle (17/26, 65%), and reduce (15/26, 58%) are covered in most studies, with remanufacture being the least covered (8/26, 31%).

#### Business Models

Most studies focused on a stand-alone value proposition (18/26, 69%). Product as a service is mentioned in 27% (7/26) of the results. The main stakeholders covered by the studies are health care professionals or hospitals (20/26, 77%), manufacturers (11/26, 42%), and consumers (9/26, 35%).

### Type of Product

The majority (17/26, 65%) of the studies have a product-specific focus, and 9/26 (35%) studies have a system-oriented focus. Out of the 17 studies that focus on a product, most of these are single-use devices (5/26, 19%) and medicine (5/26, 19%).

A more in-depth report of the results will be performed.

## Discussion

### Preliminary Findings

The review outlined will serve to understand and offer insights into the factors that influence the adoption of circular business models in health care. The findings within the micro level are expected to shed light on the product design and principles to design after. The findings within the meso level will focus on the role of stakeholders, the set of skills they must have, and key factors when collaborating. At last, the macro level will adopt broader legislation and guideline perspectives on the circular economy within health care at the national and EU levels. The findings from the review will be disseminated through a peer-reviewed scientific journal and conference presentations.

### Limitations

A total of 2 main limitations have been identified. First, as the review’s macro level focuses partially on EU legislation, there may be an overrepresentation of European studies, and the review might oversee interesting legislative initiatives in a global context. Second, in the literature search, the review only focuses on studies from 2010 onward. Therefore, older, important articles might be missed and not included in the review, which the readers should be aware of.

### Conclusion

The conclusion and outcome of the review will show that a circular business model for digital health solutions will be very specific to what kind of product it is. This will in the future require manufacturers to have a clear end-of-life management plan for their product and incorporate a layered value proposition. Furthermore, to make the value proposition and end-of-life management of the products successful, the review implies they must engage in an ecosystem with several actors to close the circular loop.

## Abbreviations

DiCE: Digital Health in Circular Economy
EU: European Union
SDG: Sustainable Development Goal

## Data Availability

All data generated or analyzed during this study are included in this published paper (and its supplementary information files).
